# Attending to Methodological Challenges in Qualitative Research to Foster Participation of Individuals with Chronic Critical Illness and Communication Impairments

**DOI:** 10.1177/23333936211000044

**Published:** 2021-04-19

**Authors:** Fuchsia Howard, Sarah Crowe, Scott Beck, Gregory Haljan

**Affiliations:** 1 The University of British Columbia, Vancouver, Canada; 2 Fraser Health, Surrey, BC, Canada

**Keywords:** communication, lived experiences, chronic, nursing, Canada, interpretive methods, qualitative methods, interviews

## Abstract

Individuals with chronic critical illness experience multiple complex physiological disturbances including ongoing respiratory failure, requiring prolonged mechanical ventilation, and thus communication impairments. In conducting a qualitative interpretive description study, we sought to ensure that individuals with chronic critical illness themselves were included as participants. Our commitment to recruiting these individuals to the study and ensuring their data meaningfully informed the analysis and findings required us to reconsider and challenge some of the traditional notions of high-quality qualitative research and develop appropriate practical strategies. These strategies included: (1) centering participant abilities and preferences, (2) adopting a flexible approach to conducting interviews, (3) engaging in a therapeutic relationship, and (4) valuing “thin” data. In this article, we extend existing literature describing the complexities of conducting research with individuals with communication impairments and strategies to consider in the hopes of informing future research with other populations historically excluded from study participation.

## Introduction

Patient experiences are an important source of evidence in nursing and health research and essential to designing and evaluating care and services. Historically, health researchers using qualitative methods have relied heavily on participants’ verbal accounts with a preference for recruiting those who can describe their experiences at length, in detail, and with particular insight (DiCicco-Bloom & Crabtree, 2006). This has not classically included those with communication impairments. The first-hand experiences of individuals with acquired impairments that affect their ability to communicate have largely been absent from the literature (Carlsson et al., 2007; Laakso et al., 2011; Lloyd et al., 2006). Such excluded individuals have been those with severe communication impairment and complex communication needs stemming from stroke, traumatic brain injury, Alzheimer’s disease, Parkinson’s disease, dementia, and neurodegenerative diseases, such as multiple sclerosis and amyotrophic lateral sclerosis. In a meta-synthesis of 293 qualitative studies of chronic illness, Thorne et al. (2002) concluded that the exclusion of individuals with diseases that influence verbal communication has rendered the diversity of chronic illness experiences invisible. When knowledge and understanding of individuals’ experiences are limited, responding adequately to their care or support needs is based on best guesses (Lloyd et al., 2006).

In this article, our purpose is to describe the methodological challenges we encountered in conducting qualitative semi-structured interviews with participants with chronic critical illness who were dependent on mechanical ventilation and residing in long-term care. The aim of the research wherein these interviews were conducted was to describe the health-related expectations and the sources of distress of residents with chronic critical illness and their families (Howard et al., 2021a, 2021b). We begin our discussion with a brief overview of chronic critical illness and our research. We then challenge some of the conventional notions of what constitutes quality in qualitative health research by describing the practical strategies we used, including (1) centering participant abilities and preferences, (2) adopting a flexible approach to conducting interviews, (3) engaging in a therapeutic relationship, and (4) valuing “thin” data. In doing so, we extend existing literature describing the complexities of conducting research with individuals with communication impairments and strategies to consider in the hope of informing future inclusive research.

## Chronic Critical Illness and Communication Impairments

In their seminal work, Nelson et al. (2010) stated that most chronic critical illness occurs among older adults who have underlying comorbidities who develop an acute critical illness when treated for a medical, surgical, neurological or cardiac illness. They defined chronic critical illness as a syndrome encompassing brain dysfunction, neuromuscular weakness, endocrinopathy, malnutrition, anasarca, skin breakdown, and symptom distress, with prolonged ventilator dependence a hallmark (Nelson et al., 2010). Though there is currently no consensus, others have included in the definition specific cut-off times for prolonged ventilation and intensive care unit length of stay that were largely based on American healthcare services and clinical contexts (Iwashyna et al., 2015). North American researchers have estimated that approximately 5% to 10% of individuals who survive an acute critical illness that is treated in an intensive care unit never recover and instead transition to chronic critical illness, with persistent dependence on life-sustaining treatments (Kahn et al., 2015; Nelson et al., 2010). Though definitions vary, individuals with chronic critical illness experience prolonged and often permanent dependence on mechanical ventilation, along with physiological, metabolic, immunological, neuroendocrine, neuromuscular, and psychological disturbances, with repeat episodes of infection and organ dysfunction (Cox, 2012; Iwashyna et al., 2015; Nelson et al., 2004, 2010; Wiencek & Winkelman, 2010).

An unintended consequence of advances in critical care treatment, chronic critical illness is an emerging and resource-intensive healthcare challenge (Lamas, 2014). The majority do not recover sufficiently to make it home but instead are discharged after an extended acute hospital stay to a dedicated weaning unit, respiratory care center, or long-term care facility (Corrado et al., 2002; Lu et al., 2012). In a 2015 meta-analysis that included studies from 16 countries, a small proportion of critically ill individuals with prolonged mechanical ventilation were discharged home from the hospital (22%, 95% CI [19–25]) and 1-year mortality was high among those in post-acute care (73%, 95% CI [67–78] in American studies and 47%, 95% CI [29–65] in non-American studies) (Damuth et al., 2015). Damuth et al. (2015) suggested that this disparity in mortality outcomes could possibly be explained by practitioners in the United States being more likely to provide a tracheostomy (a crucial step in the progression to prolonged mechanical ventilation) for patients with poor prognosis, but also variation in patient and family preferences, social acceptance of life support withdrawal, and patient/family-physician communication practices. In the United States, healthcare expenditures for this distinct population are estimated to cost $35 billion annually and long-term acute care hospitals, wherein a large majority of those with chronic critical illness are cared for, are among the fastest-growing segments of the healthcare system (Kahn et al., 2010, 2015). Though parallel economic evidence does not exist for other countries, care for individuals with chronic critical illness is a growing challenge in many developed countries (Damuth et al., 2015).

Similar to other individuals with acquired communication impairments, a large majority of individuals with chronic critical illness have deficits in one or more elements of communication. Ventilator-dependent individuals often produce speech and voice in short phrases and on both inspiration and expiration using airflow generated by the ventilator (Hoit et al., 1994). In a Swedish study, individuals whose speech output was controlled by the ventilator described achieving communication as a long and lonely struggle because of strenuous speech production and loss of speech naturalness (Laakso et al., 2011). Participants in this study characterized their voice as weak, monotonous, and difficult to vary and control, with speaking contributing to breathlessness and fatigue (Laakso et al., 2011). Furthermore, neurologic and/or cognitive impairments common among individuals with chronic critical illness can result in dysphasia, dysarthria, voice disorders, word-finding difficulties, problems in comprehending or responding to auditory information, and altered perception, attention, memory, executive function, and problem solving (Girard, 2012). Individuals with severely compromised communication might require supports in the form of augmentative and alternative communication, such as communication books or boards or voice output devices. Considering these impairments, communication can require considerable effort and energy (Fried-Oken et al., 2012). Bearing in mind their physiologic complexity and communication impairments, it is not surprising that few researchers have obtained the perspectives of ventilator-dependent individuals more generally, and of those with chronic critical illness more specifically. The majority of the research with this patient population has depended on proxy participants, primarily family surrogate decision makers, though there are a few notable exceptions (Lamas et al., 2017; Nelson et al., 2004, 2005). For example, in the study by Lamas et al. (2017), 30 of the total 50 participants were individuals with chronic critical illness who participated in semi-structured interviews.

## Our Chronic Critical Illness Research

The basis of this discussion is a qualitative, interpretive description study (Thorne, 2016) in which we interviewed individuals with chronic critical illness and ventilator dependence living in a Canadian long-term care facility. Descriptions of how this study came to be, the theoretical and methodological approach as well as study findings have been published elsewhere (Howard et al., 2021a, 2021b). In brief, clinicians and decision-makers from both the long-term care facility and the acute care hospital recognized difficulties in providing care to residents with chronic critical illness and proceeded to form a research collaboration. Through a series of collaborative meetings, these clinicians and decision-makers articulated that understanding the expectations of residents and their families, their goals of care, and their sources of distress was an important first step to improving the care and wellbeing for this population, with an end-in-view of decreasing acute exacerbations of illness and unplanned acute care readmission. Research team members were those who participated in collaborative meetings, including two critical care clinicians (a nurse practitioner and a physician with some qualitative research expertise) who provided outreach to the practice setting; two administrators from the practice setting (with minimal qualitative research expertise); two patient and family partners with related lived experience though not in the specific clinical setting (with minimal qualitative research expertise); a nurse researcher (with extensive qualitative research expertise); and a nurse research assistant (with minimal qualitative research expertise) from a university who had no clinical responsibilities at the practice setting. Thus, our research team represented diverse disciplines, expertise, clinical responsibilities, and lived experiences as well as the intended audiences for study findings.

The methodological approach of interpretive description was deemed most appropriate because it prioritizes the generating of evidence that is relevant and useful for knowledge users in the clinical setting (Thorne, 2016). Guided by an interpretive description approach, we aimed to construct subjective and experiential knowledge through interpretation and explanation that rested upon the epistemological directionality of our applied disciplines, rather than an extant theoretical framework (Thorne, 2016). We considered the reality to be complex, contextual, constructed, and subjective (Thorne et al., 2004) and aimed to articulate patterns and themes in our interpretations of the data in a manner that was relevant and useful. We also utilized integrated knowledge translation and patient-oriented research approaches, wherein all team members were considered to be equal partners in the multidisciplinary research and our aim was constructing knowledge that could be applied to improve healthcare systems, practices, and patient outcomes (Alberta SPOR SUPPORT Unit, 2018; Canadian Institute of Health Research, 2019; Kothari & Wathen, 2013). Thus, all team members collaborated during every stage of the research process from conceptualizing and designing the research, including the research purpose, sampling approach, data collection and analysis, to generating findings that would meet the knowledge needs of clinicians and decision-makers and address resident priorities, with an eye to application.

In this study, we conducted semi-structured interviews with 6 residents, 11 family members, and 21 staff. The residents with chronic critical illness who participated had heterogeneous medical diagnoses that included physiological, metabolic, immunological, neuroendocrine, neuromuscular, and psychological disturbances, and all required prolonged mechanical ventilation. Consequentially, the resident participants had varying degrees of ventilation-associated communication impairments. The interviews lasted from 45 to 150 minutes. Two team members conducted all the interviews together, one a nurse researcher experienced in conducting qualitative interviews with past experience communicating with ventilator-dependent individuals, and the other a nurse practitioner with extensive experience caring for, and communicating with, critically ill, ventilator-dependent, individuals. We obtained approval from the Fraser Health and University of British Columbia Ethics Boards for this study. We obtained verbal informed consent from all residents who chose to participate.

## Challenging Traditional Notions of Quality in Qualitative Health Research and Attending to Methodological Issues

In conducting our research on health expectations and sources of distress of individuals with chronic critical illness we were committed to trying to recruit individuals with communication impairments to the study and to ensure their data meaningfully informed the analysis and findings. This required us to reconsider and challenge some of the traditional notions of high-quality qualitative health research and to develop appropriate strategies. These strategies included: (1) centering participant abilities and preferences, (2) adopting a flexible approach to conducting interviews, (3) engaging in a therapeutic relationship, and (4) valuing “thin” data.

### Centering Participant Abilities and Preferences

Qualitative health research varies by ontology, epistemology, methodology, and purpose, but the collection of data through research interviews is common and typically involves the capturing or recording of conversations, or verbal data, that are transcribed to text (Brinkmann, 2018). Traditional notions of what constitutes a good qualitative interview in health research have largely prevailed, including in nursing research, with an emphasis on attempting “to understand the world from the participants’ point of view, to unfold the meaning of peoples’ experiences, to uncover their lived world. . .” (Brinkman & Kvale, 2018, p. 18). To understand the world from participants’ perspectives, those conducting qualitative research interviews, whether they be open-ended or semi-structured, strive to elicit “thick,” “rich,” “in-depth” descriptions of participants’ subjective experiences—detailed personal accounts of lived experiences directly related to the topic, for example (Morse & Field, 1995; Wertz et al., 2011). Despite the increasing positioning of interviews as co-constructed (Brinkmann, 2018), the ascertainment of such data is inherently heavily dependent on the abilities of those being interviewed. As such, including participants who are willing and able to share experiences in a detailed, eloquent manner is usually considered most appropriate. This is evident in the advice provided by Creswell (2013, p. 164), among others, that “the researcher needs individuals who are not hesitant to speak and share ideas. . . The less articulate, shy interviewee may present the researcher with a challenge and less than adequate data.”

Taken another way, there is the underlying and undesirable assumption that there are participants who ought not to participate in qualitative interviews. Taking this a step further, the idea of less than adequate data somewhat implies that the research participant is less than adequate and not worth interviewing. The impetus is placed on the individual participant to provide adequate data, as opposed to the researcher to co-construct data and make meaningful interpretations. In the context of communication impairments, this could further contribute to a focus on individual disability with the barrier to participation in knowledge generation residing in the individual. While the reliance on more verbose participants might result in detailed textual data, we also argue that this creates the potential to dramatically limit variability in the data and an over-representation of experiences that are easily shared, conform to socially preferred narrative forms, and demonstrate narrative resolution (see Frank, 1995 for a discussion of socially preferred narratives). Accounts that are in the midst of being formed and experiences that are difficult to articulate are in the end, simply absent. As Kirkevold and Bergland (2007) argue, the inclusion of only participants who can provide rich descriptions of their situation and experiences may generate a skewed or incomplete picture of the phenomenon of study, in which the less articulate representatives, experiences, and perceptions are left out.

Right from research conceptualization, our team challenged this notion of solely relying on participants who may be more proficient in verbally communicating these “thick,” “rich,” “in-depth” accounts. Rather, we took the perspective that we would try to recruit as many of the 22 individuals residing in the facility as possible and utilize whatever approaches and communication aids we could to capture their perspectives and co-create meaningful data in an interview. Resident communication abilities were front-and-center during the research design phase. We recognized that participants’ ventilator requirements would influence their ability to speak, with some able to speak with relative ease, some able to speak a few words at a time, and others who were non-verbal and reliant on assistive technologies, such as eye-gaze technology. This translated into our approach to data collection that centered on resident abilities rather than disabilities.

With this in mind, we planned to enhance research participation by recognizing and building on resident abilities and preferences related to the number, length, timing, and means of an interview (i.e., in-person or via email with multiple questions and answers over a longer timeframe). Because we anticipated that interviews would potentially be physically exhausting for participants, we explicitly discussed with participants a day and time that would not interfere with planned daily or weekly activities (e.g., bath day) and when they would be more likely to have energy. Based on the recommendation of Carlsson et al. (2007) to prevent participant fatigue, we offered to schedule a series of shorter interviews rather than one longer interview (e.g., 3, 30-minute interviews vs. 1, 90-minute interview). Though some initially indicated this preference, all participants were so grateful to finally be discussing their experiences that the interviews were lengthy and we, as the interviewers, were actively bringing the interviews to a close. We also discussed the inclusion of a family member or communication partner in the interview, and two interviewers participate in each interview. Our approach was congruent with those who emphasize the importance of identifying communication assets, such as speech partners, and enabling individuals to express themselves as fully as possible (Lloyd et al., 2006). In retrospect, however, our research would perhaps have benefitted from seeking the advice of people with a communication disability who are ventilator-dependent about alternate or additional strategies to center participant abilities and preferences.

### Adopting a Flexible Approach to Conducting Interviews: Conversational Partnerships and Listening, Close-Ended and Leading Questions, and Respecting Resident Self-Esteem

High-quality qualitative open-ended and semi-structured interviews in health research are often considered to be determined in part by the skill and approach of the interviewer (Brinkman & Kvale, 2018; Polit & Beck, 2017). The interviewer’s job is to encourage participants to talk freely about the research topic and to tell their stories in their own words (Polit and Beck, 2017). This positionality stems from considering the interview as primarily a research instrument that enables interviewees to describe their life experiences, rather than a social practice in itself that structures what is said and how (Brinkmann, 2018). When considering the interview as a research instrument, as is common in health and nursing research with traditions in methodologies of phenomenology and grounded theory, the focus is on what participants say with the assumption that the interview data can reflect the interviewees’ reality outside the interview (Brinkmann, 2018). As such, the role of the interviewer is to minimize their effect on how the participant describes their reality and to be as passive and non-intrusive as possible in the generation of the data (Brinkmann, 2018). The interviewer aims to create a situation wherein the participant can provide a mostly uninterrupted, well-articulated, complete picture of the situation or their experiences, and only facilitates the participant by seeking clarification of descriptions and interpretations when necessary (Kirkevold & Bergland, 2007). Kvale (1995) contended that the shorter the interviewers’ questions and the longer the participants’ answers, the better. In order to create the situation wherein the interviewer is more passive, certain interviewing techniques are encouraged, including the use of open-ended questions, limiting leading questions, and seeking clarification and using language that mirrors that of the participant (Polit & Beck, 2017). It is our observation that these techniques rely on the participant to get the interview to where it needs to go. That is, to draw attention to that which is most important and comprehensively interpret and describe the crevices and corners of their experience so that the interviewer can inductively come to know the phenomenon under study.

#### Conversational partnerships and listening

There was wide variation in the communication abilities of the participants in our study, but we were cognizant that many might have difficulty providing spontaneous and uninterrupted narratives and keeping a conversation going owing to communication abilities, neurocognitive impairments, and complex medical needs. Thus, we viewed the interviews as a research instrument, with a focus on what participants were saying, but also as the co-creation of mutual understanding that not only relied on participants to offer detailed, “rich,” “in-depth” accounts. Though we developed a traditional interview guide, our approach was to be very flexible in its use. To facilitate conversations we took direction from Bronken and Kirkevold (2013) who previously used principles from a method termed supported conversation for adults with aphasia, which was based on the idea of conversational partnerships, wherein a skilled communication partner acts as a resource for the participant by using communication techniques such as active listening, prompting, helping with word retrieval, writing down keywords, and offering alternatives and validation (Kagan, 1998). We discovered the tremendous value of having two interviewers conduct each interview, both of whom were experienced in communicating with ventilator-dependent individuals. Having two interviewers enabled us to understand and focus on the content and meaning of participant responses and ensure participant responses were accurately captured.

We also found it helpful to acknowledge and honor the time, energy, patience, and concentration necessary to listen carefully and thoughtfully stay “with” the participant and respond accordingly (Morse & Field, 1995). With two interviewers, we could follow up for clarification of our understanding or interpretation to a greater extent. Others have argued that it is the response of those who do not listen carefully enough that transforms a communication difficulty into a disability (Cheston, 2000) and so we put tremendous effort into listening carefully.

Our ability to listen and understand was further enhanced in some interviews wherein a family member participated and functioned as a communication partner. In the following transcript excerpt, the interviewer posed a question to the resident whose speech was typified by short phrases wherein their voice trailed off as they spoke (at the end of expiration). Though the interviewer attempted to reiterate what they thought the resident had said, the family member was key to interpreting and restating the resident’s words and then pausing for the resident to add to the phrase and complete their thought. When the resident’s tears interfered with their ability to speak, the family coached the resident to take a “deep breath”—enhancing the resident’s speech, but also providing emotional reassurance and encouragement to continue. Moreover, the family member double checked with the resident to confirm that the co-constructed phrase communicated what the resident intended.

Interviewer: One of the other things that some of the residents have said is that it can be really hard emotionally, and there can be sadness. But others have said, you know, it’s okay or it comes and goes. What has been your experience, [resident name]?

Resident: Comes and goes.

Interviewer: Comes and goes. And so what are the good days like for you?

Resident: Like today.

Family: Like today? Is that what you said?

Resident: Sleep, go outside, come back, and watch TV and go to sleep. That’s all. . . [inaudible and patient begins to cry]

Interviewer: That’s all you can do. If—sorry?

Family: Deep breath.

Resident: Bad days—

Family: Bad days.

Resident: —are ones that I [inaudible]. She’s [inaudible].

Family: Okay, bad days—

Interviewer: Your wife?

Family: —is when—?

Resident: When [wife] says—

Family: When [wife] says—

Resident: —she’s not with me anymore

Family: She—?

Resident: [inaudible]

Family: She’s not with you anymore? Because I think his wife’s having a struggle too, so.

Interviewer: I’m sorry. That must be really hard.

Resident: That’s tough.

Interviewer: Yeah.

Family: Mm-hmm.

Despite the benefit of having a family member assist the resident to communicate, we were also cognizant of our need to balance the communication such that the family member did not dominate the process of communication. Strategies we used included using participants’ names and non-verbal body language (e.g., eye contact) to direct questions to either the resident or the family member specifically. While we centered the interview and the interview questions on resident experiences, we also intentionally created space for family members to discuss their own experiences, including reflecting on their own sources of distress and health-related expectations of the resident, as apparent in the following exchange between an interviewer, resident, and family member.

Interviewer: What other goals are the most important for you, [resident name]?

Resident: Uh, [pause and looks to family member]

Family: I think I’ve always set our sights on right now. I mean once you can get to that level, maybe there’ll be new goals. But, we need to, you know we’ve set out goals for now, to get her in a chair. And, interacting more and outside more, and,

Resident: Yeah.

Family: And then when we reach that goal then we’ll set the next one.

Interviewer: So a bit of a hard question. But what are your biggest fears and worries [resident name], with regard to your health?

Resident: My biggest worry, is I won’t get out of here. I won’t get better. But I don’t want to be a burden on my [family member]. I think that’s, the biggest one.

Interviewer: And [family name] what about for you? What are your biggest fears and worries for your [family member]?

Family: That she’s not happy. That she’s enduring for me. And she’s putting on a brave face for me. [laughter] I wasn’t gonna cry! [crying] And I mean I have to say it and we’ve talked about it. So I can say it. You know that one infection is gonna be bad enough and, just, she won’t recover from it. We’ve had a couple of good scares already and we know that she’s got a colony of bugs in her that will never go away. They’re there. And they’re dormant in her body and it’s just a matter of, do they flare up again? Or do they stay dormant? So that’s my fear.

Resident: Yeah, I know.

Interviewer: And, in the event that you get sick again, how much and what, what else are you willing to endure?

Resident: Uh, I’ve been thinking about this, lately, you know I haven’t talked about it with you [family name], but, what point do I want to live? What point, do I wanna be [inaudible] do I? Wanna have no, [inaudible]

Family: No resuscitation?

Across interviews, and evident in the above quote, creating opportunities for family members to also share their own experiences further facilitated and encouraged resident sharing.

#### Close-ended and leading questions

In some interviews, we also adopted techniques traditionally considered to be characteristics of poor interviews, including the use of closed-ended, simplified or leading questions, and paraphrasing (Morse & Field, 1995). In some instances, we modified the wording of questions and prompts to facilitate shorter, less wordy answers that allowed simple responses. This is evident in the following transcript exert wherein the interviewers posed questions that asked for yes or no responses, or for the resident to choose one of the options offered. Both interviewers also paraphrased the resident’s responses at different times and followed up with leading questions based on their interpretation of the resident’s experiences.

Interviewer 1: When you think about your fears and worries for the future, what are the big ones, for you? Around your health, but also around your care.

Resident: [inaudible] don’t.

Interviewer 1: You don’t know. Would you fear, getting ill and going to the hospital more? Or would you fear staying where you are more?

Resident: Staying here [facility].

Interviewer 2: Staying here [facility] is worse than going back to the hospital.

Interviewer 1: The last time you went to the hospital, did you feel like you knew you were sick before, before the staff picked up on it or not?

Resident: Yes.

Interviewer 1: Okay.

Interviewer 2: Do they listen when you say I’m sick and I need to go [to the hospital]?

Resident: No.

Interviewer 1: No.

Resident: It’s all about, [inaudible] symptoms.

Interviewer 1: About finding symptoms?

Resident: It’s all about, [inaudible]

Interviewer 2: Oh, they say you don’t have any symptoms. And they’re trying to convince you not to go? But you feel sick. And you know yourself better. Is that what you are saying?

Resident: [nodded head yes] I’ve told them, a million times, listen [inaudible passage] I know.

Interviewer 1: So you get worse instead of better. And what’s their reaction when you say that? Do they listen or ignore you?

Resident: Ignore.

Our approach is at odds with and challenges those who contend that researchers should try to limit their shaping of the data. For example, Morse & Richards (2002) argued that qualitative interview data may be of lower quality when the researcher has too much of a presence in the data. However, we concur with others that this form of more simplified and at times, use of leading interview questions was justified on the grounds that it supported the process of generating relevant data and allowed contributions from individuals who otherwise would be excluded from study participation (Bronken & Kirkevold, 2013; Philpin et al., 2005).

#### Respecting resident self-esteem

Finally, in our flexible approach to interviewing we took steps to prevent the unnecessary highlighting or exposing of participant disabilities that could lower self-esteem and self-worth. In a study of stroke patients, Kvigne et al. (2002) suggested that changes in appearance and bodily perception, reduced memory, and loss of linguistic and physical skills can intensify the participants’ feelings of powerlessness. They further contended that a feeling of diminished self-image, low self-esteem, and self-worth can lower an individuals’ faith in their own message and lead to the omission of important messages and socially unacceptable details. Building on this idea in reference to participants with memory deficits, Kirkevold and Bergland (2007) stipulated that it is mandatory to avoid questions that expose problems or deficits in a way that threatens the integrity of the person.

In our research, we were cognizant of not only communication impairments but also neurocognitive difficulties experienced by individuals with chronic critical illness and our ethical imperative to respect resident self-esteem and self-worth. We anticipated that interviews could potentially be anxiety-provoking owing to participant embarrassment and worry that their memory was flawed and that they would “not have the right answers.” As such, when constructing the interview guide we avoided questions that highlighted memory impairments, such as, How long have you lived here? Or when was the last time you were re-admitted to the hospital? At the outset and throughout the interviews, we focused on building rapport and trust by providing ongoing reassurance that there was no right or wrong answer and that the participants’ perspectives and experiences were important and of value to the research. In the following excerpt from the beginning of an interview, the resident indicated that they did not want to talk about their understanding of where they were at with their health. Recognizing that the resident was perhaps interpreting this question to be the request for detailed medical questions, the interviewer provided clarification that facts were not being requested and reassurance that they could not answer the question incorrectly. The interviewer then checked in to see whether the resident was comfortable to proceed with discussing how they felt about their health.

Interviewer: So, what is your understanding now, of where you are with your illness, your medical condition, your health? In general?

Resident: Not gonna go there.

Interviewer: Okay. That’s okay. Just so you know, there’s no right or wrong answers. We’re not looking for you to say, oh I have this condition, or that condition. It’s more about where you feel you’re at with your health. Would that be something you’re willing to talk about?

Resident: [nodded yes]

Interviewer: Yeah?

Resident: [nodded yes]

Interviewer: Okay.

Moreover, in instances where there were inconsistencies in participants’ accounts, related to timelines or treatment details, for example, we intentionally did not seek clarification or attempt to determine the “right answer.” We also gathered medical and sociodemographic data from the resident medical charts and ensured residents had the option of conducting the interview with a family member of their choice. The participants who opted to include a family member in their interview often deferred to their family member to recall experiences surrounding their medical history or instances of acute exacerbation of illness. Despite our use of strategies to reduce the risk of lowering participant self-esteem and self-worth, seeking the advice of people with a communication disability who are ventilator-dependent about alternate or additional strategies would again, likely be beneficial.

### Engaging in a Therapeutic Relationship

The goal of a qualitative interview in health research, including in nursing, is often considered to differ from that of a therapeutic interview. Awareness of this difference likely stems, in part, from the safety plans researchers develop to obtain permission to conduct research from an Institutional Review Board. These safety plans are developed out of the recognition that participating in research about sensitive topics or experiences might carry an emotional risk wherein participants relive difficult emotions. To minimize emotional risks, health researchers often include the following as ways to respond to difficult emotions during an interview: (a) waiting for the participant to collect themselves before continuing; (b) asking the participant if they would prefer to stop the interview; (c) redirecting the interview to a less emotionally laden topic; or (d) referring the participant to a professional counselor (DeMarrais & Tisdale, 2002). Though minimizing emotional risks is essential, this can also be taken up as minimizing engagement in the therapeutic aspects of an interview.

The qualitative interviewer seeks to elicit information and meaning, learn from participants, and come to understand the phenomenon of study. In contrast, clinical, therapeutic interviews are meant to engender change and be of benefit to the patient (Targum, 2011). In qualitative interviewing, it is the researcher who seeks out participants who can provide data for the study, while in therapy, a distressed patient seeks a counselor who will direct the client toward a healthier and more functional life (DeMarrais & Tisdale, 2002). Rossetto (2014) also argued that qualitative interviewees share events, situations, and information relevant to the phenomenon of study, while therapeutic interviewees discuss internal states and process previous sources of these states. The researcher listens intensely and provides space for the participant to share their experiences, while the therapist interprets the stories for the purpose of ameliorating distress (DeMarrais & Tisdale, 2002). Further, the relationship between the interviewee and the researcher or therapist is considered to be different in that researchers contribute to the co-production of meaning, while therapists are authority figures, responsible for life changes and improvement by enabling affective experiencing, cognitive mastery, and behavioral regulation (Rossetto, 2014).

Despite understanding the different goals of qualitative and therapeutic interviews, this distinction between the two became somewhat blurred in interviewing individuals with chronic critical illness in our study. All of the participants and several family members shared deeply personal accounts and expressed emotions such as sadness, despair, worry, regret, anger, fear, hope, and gratitude in the interviews. Some of the participants shared personal and intimate stories and experiences that they had not shared previously, including with family members, some of whom were also present in the interview.

Interviewer: So, I would like to hear more about where you see yourself going.

Resident: Have I said too much? [question addressed to both the interviewer and family member]

Interviewer: No, not at all. I really very much appreciate your sharing.

Resident: What I’ve said is hard to say. I think. [pause and tears up] You know why? If I, it was just me here, that’s one thing. It’s tough to say in front of you, [family member]. Cause it’s more than I’ve told you. Yes?

Family: Yes. But we’re, we’re doin’ this together. And I want to know what it’s like. What you, how it is for you too, to get through this.

Most of the participants cried at some point during the interview. In reflecting on the interviews and the study findings that residents experienced deep sadness and often felt alone, neglected and frustrated with few opportunities to engage in meaningful activities (Howard et al., 2021a), it is not surprising that these interviews seemed to engender a therapeutic element for the participants. Considering that social interactions of the residents were by-and-large confined to staff and family, the interviews were perhaps an opportunity to engage in a confidential conversation about their challenges as they perceived them and with interviewers who were not involved in their care. We also broached difficult topics of conversation that residents and family had not always discussed.

While we were aware that the interviews might be emotional and accordingly developed a plan if participants became distressed, we were struck by our ethical obligation to attend to the well-being of participants throughout the interview process. During the interviews, the participants shared deeply personal and sensitive information. We quickly came to understand the intimate nature of these interviews and the tension between wearing our nursing/therapeutic hats versus wearing our researcher/data co-production hats.

As nurse researchers conducting the interviews, we could not shy away from, nor discourage, the sharing of deeply personal and emotional experiences, but rather, embraced that the interviews could be therapeutic. That is, we were clear that our intent was not to provide therapy, but that research participation could yield therapeutic benefits through engagement in a therapeutic relationship. With this in mind, it was essential that we conveyed caring, compassion, and empathy both verbally (e.g., by acknowledging emotions, providing reassurances, paraphrasing, and gently probing) and non-verbally (e.g., by holding or touching a participants’ shoulder or hand, maintaining eye contact, and an open body position), and encouraged the sharing of emotions (e.g., by talking through the issues). This also meant being open to where the participants’ wanted to go emotionally during the interview and acknowledge their pain and suffering. The presence of two interviewers in each interview further facilitated the picking up of the more nuanced verbal and non-verbal signs of sensitive or emotional topics that we might otherwise have missed. We align with others who view qualitative interviews as potentially therapeutic (Birch & Miller, 2000; Dempsey et al., 2016; Eide & Kahn, 2008; Holloway & Wheeler, 1995; Shamai, 2003), wherein the term therapeutic is used to represent an emotional process wherein the participant reflects on and comes to understand previous experiences in different ways, promoting a changed sense of self with new understanding. According to Shamai (2003), a therapeutic relationship is founded on empathic listening, witnessing the expression of emotions and the disclosure of a private self, and acknowledging the participant’s experience and self-worth. Reflecting on our study, we concur with Rossetto (2014) that researchers must acknowledge the therapeutic possibility of the qualitative interview because it can and should affect participants’ reactions, interviewers’ approaches, and how researchers can make a difference in people’s lives.

Despite viewing the interview as engaging in a therapeutic relationship, we also recognized the need to maintain boundaries to protect the researcher-participant relationship and our ethical obligation to do no harm (Birch & Miller, 2000; Dickson-Swift et al., 2006). After all, we were bound by the procedures stipulated in our approved research ethics application. We were cognizant of the ethical imperative to conduct the interview in a manner that aligned with study goals, as conveyed during the informed consent process, as well as support participant self-determination. We tried to maintain the purpose of the interview as coming to understand the participant’s emotional world, rather than pursuing a line of questioning or thought for therapeutic effect. We were also particularly sensitive to recognizing signs of distress and pulling back if participant responses became negative. When participants became upset, we asked if they would like a break from the interview, re-directed the interview if they wished, and were prepared to refer them to a counsellor if desired. Despite the presumed dichotomy between researcher and therapist, we found the practice of conducting these highly emotional interviews as nurse researchers was not straight forward. Rather, this demanded the constant encouragement of thoughtful and personal sharing, while also trying to obtain information relevant to the study, monitoring for emotional distress, maintaining boundaries, and abiding by our ethical research obligations. This involved thoughtful gauging and re-calibrating throughout each of the interviews.

### Valuing of “Thin” Data

In our attempt to be inclusive of participants with a range of abilities to provide detailed accounts and the use of non-conventional interviewing approaches, some of our interview data was rather “thin” by conventional qualitative health research standards, as exemplified by the following transcript excerpt.

Interviewer: What makes a bad day?

Resident: The nurses.

Interviewer: The nurses. And what are the types of things that they do to make it bad?

Resident: [Long breath] it’s either their way or [mouthed the word nothing]

Interviewer: Their way or nothing?

Resident: [inaudible]

Interviewer: So what types of things? Can you give me an example?

Resident: Everything and anything. [long breath] so that you have to. [long breath] it’s a vicious setting.

Interviewer: It sounds incredibly frustrating. Is there anyone you talk to about that?

Resident: [participant nodded head no]

Interviewer: No?

Resident: It would get back to the nurses.

Interviewer: And what would happen if it got back to the nurses?

Resident: I’ll get the look.

Interviewer: You’ll get a look?

Resident: You did it.

Interviewer: A look of, I know you did it? And is that? Correct me if I’m wrong, but to me it sounds like a form of punishment?

Resident: It is. I get the silent treatment. It’s really hard and it’s [long breath], “you did it.” [long breath]. Cause they’re [the nurses] mad at me. [long breath] over and over and over.

During data analysis, we were cognizant that to ensure this “thin” data informed our interpretations we had to approach data analysis more narratively rather than thematically dissecting the data. This meant trying to ensure we were interpreting the larger narratives and participants’ intended meanings rather than relying on line-by-line or even broader dissection of the data. This analysis demanded re-reading of whole transcripts rather than relying on data that had been fractured and extracted through thematic coding. As analysis proceeded, we also moved away from the literal or staying close to the data, to a more abstract, interpretive stance. This approach contrasts with a growing emphasis in qualitative health research on “theme” identification as a legitimate endpoint of analysis, rather than more interpretive findings that add insight (Thorne, 2020). The liberty of interpretation we deemed appropriate and aimed for was supported by drawing extensively on the relevant knowledge of our clinician team members.

In our interpretations and textual presentation of our findings we were aware of the potential for this seemingly “thin” data, and the corresponding experience of individuals with more severe communication impairments, to be relegated to the sidelines. The ethical imperative to “give voice” to participants is often met through long, detailed block quotes that provide evidence of authentic, credible research. The majority of approaches to appraise the rigor of qualitative health research findings suggest that authors should provide evidence (e.g., examples, quotes, or text excerpts) to substantiate the main analytic findings (O’Brien et al., 2014; Tong et al., 2012). It is our perception that in the health sciences, including nursing research, this evidence has largely been taken up to mean thick, rich, vivid descriptions that include verbatim quotes from study participants.

In presenting our findings we struggled, at times, to illustrate the point we were making through the inclusion of block quotes from participants with communication impairments. When block quotes were unavailable, and to ensure we were not only reliant on the participants who were more easily understood, we integrated phrases or words used by the participants throughout our written findings and summarized examples provided by participants. Even then, reviewers of our manuscripts reporting study findings requested additional participant stand-alone, block quotes. This raised for us the tension between “giving voice” and the value of qualitative interpretation in articulating experiences in a manner that conveys meaning as intended by participants. In discussing their qualitative research with persons with aphasia, Bronken and Kirkevold (2013), contend that the researcher’s interpreted descriptions might be closer to the participants’ meaning than what they term language-impaired quotes. Furthermore, they posed several important questions of relevance to our research, including: How rich do participants’ accounts need to be to be considered rich enough? What does rich enough really mean? Does relevant thin data compensate for the threat that would result from exclusion specific to sampling bias? These contentious questions challenge us as researchers to consider the implications of only valuing traditional conceptualizations of quality interview data and implore us to be thoughtful in the development of strategies that enable the meaningful inclusion of other forms of data and the importance of interpretation. And yet, this also brings into focus what Brinkmann (2018) refers to as the interviewer’s monopoly of interpretation, where the researcher, as the “Big Interpreter,” has the exclusive privilege to interpret and report what the interviewee really meant.

## Discussion

We were motivated to conduct this research in part because of the invisibility, both in the social world but also in the academic literature, of individuals with chronic critical illness and the tremendous suffering they endure. While this research poses practical challenges that must be considered and addressed, developing means of capturing the personal perspectives of these individuals whose accounts have historically been excluded also represents a form of validation, empowerment (Lloyd et al., 2006), and social inclusivity. With a patient-centered healthcare services goal in mind, including people with a chronic critical illness in research is also necessary to gain insight into their experiences as a foundational step to meeting their care needs.

Practical strategies we found helpful to conduct semi-structured qualitative interviews with individuals with chronic critical illness included centering participant abilities and preferences rather than their disabilities when planning and conducting interviews and adopting a flexible approach to conducting interviews. This included facilitating interviews through the inclusion of conversational partnerships, focussing on listening, using close-ended and leading questions at times, and taking steps to respect participant self-esteem. We also found it important to view the interviews as engaging in a therapeutic relationship, yet recognized our need to balance this with our ethical imperative to conduct the interviews in a manner that aligned with study goals. Lastly, we found it helpful to question notions of “thin” data during our analysis and interpretations of participants’ accounts.

Our research adds to the growing body of evidence constructed through qualitative studies that include individuals with a communication and/or neurocognitive impairment as interview participants (Bronken & Kirkevold, 2013; Carlsson et al., 2007; Carroll, 2007; Cheston, 2000; Kagan, 1998; Kirkevold & Bergland, 2007; Kvigne et al., 2002; Laakso et al., 2011; Lamas et al., 2017; Lindahl et al., 2006; Lloyd et al., 2006; Nelson et al., 2005; Philpin et al., 2005; Sakellariou et al., 2013; Teachman et al., 2018). Some of the strategies we used to facilitate research participation were similar to those previously described. We drew heavily on our knowledge and skills as healthcare providers in nursing and medicine to make space for listening to, and understanding study participants. Like others (Carlsson et al., 2007; Carroll, 2007), having sufficient knowledge about the nature of participants’ impairments, its effects on the interviewing process and the skills to engage were particularly helpful. Our clinical orientation also directed us to be flexible, oriented to the individual, and engage therapeutically while conducting our research. Dickson-Swift et al. (2006) considered the differentiation between qualitative research interviews and therapy or counseling interviews to be a boundary blurring issue. They contended that research often involves the disclosure of intimate and private aspects of participants’ lives, which could mirror the position of a therapist. As such, they recommended that researchers be aware of the possibility of the blurring of the researcher-therapist boundaries and accordingly have strategies in place, including the use of research supervisors to facilitate discussions and planning for researcher self-care. In addition to the explicit recognition that researchers engage therapeutically with participants, future efforts to identify and develop strategies for doing so would be of benefit. It is simply unhelpful to pretend this boundary blurring does not exist.

Our approach of conversational partnerships, informed by the work of Bronken & Kirkevold (2013) and Kagan (1998), was also useful but has implications for how we might view and interpret such data, similar to data generated through joint interviews. Norlyk et al. (2016) argue that the presence of a partner in a joint interview favors expressions of shared rather than individual experiences. For example, in their research on motor neuron disease/amyotrophic lateral sclerosis, Sakellariou et al. (2013) reflected that even though individual perspectives were expressed in joint interviews, these individual perspectives were sometimes difficult to discern because the stories became intertwined, “one entering onto the other.” (p. 1568) This co-construction of meaning becomes that much more obvious when participants have communication impairments.

In reviewing qualitative interview research Lloyd et al. (2006) suggested that, “it is possible to elicit perspectives and experiences verbally from individuals with expressive language difficulties and that even in situations where this is accompanied by progressive cognitive impairment, a voice can still be found” (p. 1388). In reflecting on our study and the strategies we used to include individuals with chronic critical illness as interview participants, we grew increasingly uncomfortable with the language of “giving voice” as a goal of qualitative research. The intention to “give voice” through qualitative research seems to imply mining for voices that simply exist (Facca et al., 2020) and then acting as a megaphone for participants in the ethical service of democratizing knowledge (Brinkmann, 2018). In their research with disabled youth who use augmentative and alternative communication, Teachman et al. (2018) critique dominant notions of voice, suggesting that “voice only exists in the relation *between* two or more speakers in the context of talk,” and is, thus, “not an individual property that researchers can retrieve, enable and possess through interviews” (p. 38). We came to understand that our role was not to “give voice,” but rather, engage with and listen to individuals who are rarely heard, to explicitly acknowledge our role plus that of family members in co-constructing meaning, and to convey through findings, our interpretation of participants’ experiences and perspectives. Moreover, these interpretations were intended to be useful to our research team, not necessarily to participants themselves, in generating clinically meaningful knowledge that was relevant and useful in the clinical context.

## Conclusion

We anticipated and encountered challenges conducting our research, yet these challenges were surmountable with a team who was willing to be flexible and responsive to participant abilities, were highly skilled in qualitative interviewing and communication with individuals dependent on a ventilator, and embraced engagement in a therapeutic process while also being cognizant of ethical research obligations. This research pushed us to consider and question traditional notions of quality interviewing and what constitutes “good data.” The development of quality criteria for evaluating qualitative research, while an important step forward, must not be blindly applied without consideration of the study context and all that is involved or intentionally done by the research team.
